# Physical exercise and depressive symptoms among rural older adults in China: a cross-sectional study of serial correlational linkages via health anxiety and self-efficacy

**DOI:** 10.3389/fpsyg.2026.1940756

**Published:** 2026-10-26

**Authors:** Jian Jiao, Xianzhong Chen

**Affiliations:** 1 School of Physical Education and Sports, Shenzhen University of Information Technology, Shenzhen, China; 2 Department of Physical Education, Shenzhen Polytechnic University, Shenzhen, China

**Keywords:** aged, depressive symptoms, health anxiety, rural population, self-efficacy

## Abstract

**Background:**

China has entered a stage of deep population aging. The relatively high detection rate of depressive symptoms among rural older adults has become a prominent challenge in grassroots elderly health governance. As a modifiable lifestyle factor, physical exercise is widely associated with lower depressive symptoms, yet research on the localized associative pathways underlying this correlation targeting this specific group remains insufficient. This study examines the specific and serial indirect associations of health anxiety and self-efficacy in the association between physical exercise and depressive symptoms, to provide empirical evidence for developing potential intervention strategies among rural older adults.

**Methods:**

Employing a combination of multi-stage stratified random sampling and quota sampling, this study selected 772 rural older adults aged 60–69 years from eastern, central and western regions of China as the research sample. Data were collected through home visits using standardized scales. The Bootstrap method implemented in SPSS 27.0 and the PROCESS 4.0 macro was applied to estimate the indirect associations.

**Results:**

Physical exercise showed a significant negative cross-sectional correlation with depressive symptoms, with a total association estimate of −0.0793 (95% CI: [−0.0992, −0.0595]). The direct association was −0.0356 (95% CI: [−0.0566, −0.0147]), accounting for 44.89% of the total association. The total indirect association of health anxiety and self-efficacy was −0.0437 (95% CI: [−0.0555, −0.0331]), contributing 55.11% to the total association. All three indirect paths were statistically significant: the specific indirect association via health anxiety was −0.0266 (95% CI: [−0.0362, −0.0180]), accounting for 33.54% of the total association; the specific indirect association via self-efficacy was −0.0115 (95% CI: [−0.0183, −0.0060]), accounting for 14.50% of the total association; and the serial indirect association was −0.0057 (95% CI: [−0.0087, −0.0032]), accounting for 7.07% of the total association.

**Conclusion:**

Higher physical exercise levels correlated significantly with milder depressive symptoms in this cross-sectional sample of rural Chinese older adults. Health anxiety and self-efficacy showed specific indirect associations within the physical exercise–depressive symptom association, including a serial indirect association through both constructs. Specifically, health anxiety occupied the most prominent position within the observed network of inter-variable correlations.

## 1 Introduction

Depressive symptoms are an important concern among rural older adults in China, where persistent urban–rural disparities in symptom prevalence have been reported (Yu et al., 2023). Research on exercise and depressive symptoms in older adults provides a rationale for examining physical exercise in this population (Lili et al., 2024). However, the joint pattern of associations involving physical exercise, health anxiety, self-efficacy, and depressive symptoms remains insufficiently characterized among rural Chinese adults aged 60–69 years.

Health anxiety concerns the interpretation of bodily sensations or changes as signs of serious illness (Asmundson et al., 2010). Self-efficacy refers to beliefs about one’s ability to cope with challenges. These constructs capture potentially relevant aspects of perceived threat and coping capacity. We hypothesize that higher physical exercise scores are associated with lower health anxiety and higher self-efficacy, and that these two constructs are, respectively, positively and negatively associated with depressive symptoms. The proposed associations provide a rationale for testing specific statistical indirect associations; they do not establish how these variables influence one another.

We further hypothesize a negative association between health anxiety and self-efficacy. Together with the proposed associations above, this would be consistent with a serial indirect association ordered as physical exercise, health anxiety, self-efficacy, and depressive symptoms. This ordering is a model assumption requiring evaluation, rather than an established temporal or causal sequence.

The present cross-sectional study examines these associations among rural adults aged 60–69 years recruited from eastern, central, and western China. It tests an inverse association between physical exercise and depressive symptoms (H1), specific indirect associations through health anxiety (H2) and self-efficacy (H3), and a serial indirect association through both constructs in the hypothesized order (H4). Physical exercise is represented by the continuous PARS-3 score in these analyses, and depressive symptoms refer to CES-D 10 scores rather than a clinical diagnosis. The study evaluates statistical associations and cannot establish causal effects or temporal mediation.

## 2 Literature review and hypotheses

### 2.1 Physical exercise and depressive symptoms among rural older adults in China

As China’s population aging continues to intensify, the mental health risks of rural older adults have become increasingly prominent. Depressive symptoms in this group are associated with poorer perceived quality of later life and may co-occur with reduced physical function and cognitive capacity, making symptom monitoring an important concern in rural elderly health promotion initiatives (Qing et al., 2024). Identifying correlates of fewer depressive symptoms carries important practical value for building a low-cost, widely accessible intervention system for rural elderly mental health.

Existing empirical evidence indicates that physical exercise is inversely associated with depressive symptoms (Zhao et al., 2025). Potential physiological explanations include associations between regular physical exercise and neurotransmitter regulation, hypothalamic–pituitary–adrenal (HPA) axis responsivity, and systemic inflammation; these explanations require longitudinal or experimental evaluation (Xue et al., 2025). Potential psychological explanations include associations between physical exercise, positive emotional experiences, attentional patterns, and perceived social support; the cross-sectional design does not establish these as causal pathways (Arent et al., 2000).

Compared with their urban counterparts, rural older adults generally face realistic constraints such as limited accessibility to medical resources, insufficient provision of formal social support, and emotional absence caused by intergenerational separation, resulting in multiple correlates of depressive symptoms. Although this group engages in a certain degree of daily productive physical activity, their participation in structured and regular physical exercise remains generally low, and the inverse association between physical exercise and depressive symptom scores in this specific population still requires targeted verification. Based on the theoretical framework of health promotion and logical deduction from existing research conclusions, physical exercise is expected to show an inverse cross-sectional association with depressive symptom scores among rural older adults. Accordingly, the following research hypothesis is proposed:

*H1*: Physical exercise will show a statistically significant inverse correlation with depressive symptom severity among rural older adults in China. The model hypothesizes that higher self-reported physical exercise volume will correlate with lower depressive symptom scores in cross-sectional observation.

### 2.2 Specific indirect association through health anxiety

Existing studies have reported an inverse association between physical exercise and depressive symptoms among older adults, yet the statistical pathway underlying this association among rural older adults in China remains to be further elucidated. As a core variable reflecting individual health-related cognitive bias, health anxiety may represent a candidate intermediary in the observed association between physical exercise and depressive symptoms (Ding et al., 2026; Song et al., 2025).

Cross-sectional correlational studies report inverse links between physical exercise and health anxiety. From a theoretical physiological perspective, regular exercise may correlate with slower age-related physical function decline, better self-reported health status, and fewer somatic complaints; these somatic indicators are further hypothesized to correlate with lower subjective health worry (Romero et al., 2025). Cognitively, the sense of body control and experience of health improvement gained during exercise can correct individuals’ catastrophic cognition of health risks, and reduce excessive attention and negative interpretation of somatic symptoms (Oh and Park, 2025). Compared with urban older adults, rural older adults have relatively insufficient health literacy and poorer access to primary medical resources, making them more prone to negative and extreme attributions of somatic discomfort. Their health anxiety is notably more responsive to behavioral interventions, lending greater practical relevance to the alleviating statistical association of physical exercise on health anxiety in this population.

On the other hand, health anxiety is positively associated with depressive symptoms in older adults. According to Cognitive Behavioral Theory (CBT), persistent health anxiety traps individuals in repetitive worry about illness and excessive awareness of somatic symptoms, which continuously reinforces the negative belief of loss of health control and co-occurs with depressive symptoms such as low mood and diminished interest (Asmundson et al., 2010). Rural older adults are undergoing a transitional phase marked by social role shifts and physical function decline. The sense of helplessness and uncertainty induced by health anxiety is more likely to develop into persistent depressive mood, making health anxiety a relevant correlate of depressive symptom severity in this group. The observed data are therefore compatible with both a direct association and a specific indirect association through health anxiety, but they do not establish that physical exercise reduces depressive symptoms by reducing health anxiety. Accordingly, the following research hypothesis is proposed:

*H2*: Health anxiety will show a specific indirect association within the cross-sectional association between physical exercise and depressive symptoms among rural older adults in China.

### 2.3 Specific indirect association through self-efficacy

In line with the core logic of Bandura’s Social Cognitive Theory (SCT), self-efficacy—defined as individuals’ subjective beliefs about their ability to accomplish specific tasks and cope with real-life adversities—may function as a candidate intermediary variable in the observed association between behavior and mental health status (Cahill and Bandura, 1987). Its potential statistical role in the association between physical exercise and depressive symptoms among rural older adults can be examined without assuming a one-way transmission pathway (Guoyan et al., 2023).

For rural older adults in China, regular physical exercise may be associated with higher self-efficacy through multiple correlates. Mastery experiences derived from improved physical function and the achievement of phased exercise goals constitute the most direct source of self-efficacy improvement (Bandura, 1977). Informal social support and positive peer feedback generated through group exercise interactions in rural community settings can further strengthen older adults’ positive perceptions of their physical regulation ability and social adaptability, gradually alleviating the sense of diminished competence that emerges during natural aging, and may therefore coincide with higher general self-efficacy and emotion-regulation self-efficacy (Yu et al., 2025).

Meanwhile, self-efficacy is inversely associated with depressive symptoms in older adults. Rural older adults generally face multiple realistic stressors, including declining physical function, shifting family roles, and insufficient access to primary medical resources. Individuals with high self-efficacy are more inclined to adopt problem-focused positive coping strategies to address life difficulties, reduce negative cognitive rumination, and lower the likelihood of helplessness and hopelessness, and may therefore coincide with fewer depressive symptoms (Raphaël et al., 2009). The observed inverse association between physical exercise and depressive symptoms may therefore include both a direct association and a specific indirect association through self-efficacy; the cross-sectional data do not establish that exercise elevates self-efficacy or that self-efficacy subsequently reduces symptoms. Accordingly, the following research hypothesis is proposed:

*H3*: Self-efficacy will show a specific indirect association within the inverse cross-sectional association between physical exercise and depressive symptoms among rural older adults in China.

### 2.4 Serial indirect association through health anxiety and self-efficacy

Grounded in Social Cognitive Theory (SCT) and the cognitive-behavioral model of health anxiety, and in consideration of the health ecological characteristics of rural older adults in China, this study proposes the research hypothesis that health anxiety and self-efficacy form a hypothesized serial indirect association within the inverse cross-sectional association between physical exercise and depressive symptoms. Regular physical exercise may be associated with lower health anxiety through physical-function indicators and mastery experiences reported during exercise. Rural older adults often face risks of chronic strain and underlying chronic diseases, coupled with relatively limited access to primary medical resources; they are thus prone to developing catastrophic cognition toward daily somatic discomfort, which may co-occur with excessive health concerns. Sustained physical exercise may be associated with better cardiopulmonary function and physical tolerance, and with fewer somatic complaints (Goodarzi et al., 2025). Meanwhile, better physical condition may coincide with less anticipation of health risks and lower health-anxiety scores (Jin et al., 2026).

Higher health anxiety is also associated with lower self-efficacy. Individuals with high levels of health anxiety tend to selectively attend to negative somatic signals, overestimate health threats while underestimating their own coping capacity, forming a negative cycle of somatic discomfort and cognitive worry (Zhu et al., 2025). For rural older adults, health status is directly associated with their fulfillment of family roles and maintenance of daily self-care ability. Persistent health anxiety may coincide with lower confidence in life control and problem-solving, and with lower general self-efficacy (Park, 2024). Self-efficacy is inversely associated with depressive symptom severity in older adults. Its level is associated with coping styles and emotion-regulation abilities when individuals face stressful events. Lower self-efficacy may co-occur with learned helplessness and depressive mood (Caitlin et al., 2020). Among rural older adults, self-efficacy is largely based on daily activity competence and social role participation. Lower self-efficacy may be associated with reduced emotional resilience and greater depressive symptom severity (Zuo and Chen, 2025). Accordingly, the following research hypothesis is proposed:

*H4*: Health anxiety and self-efficacy will show a serial indirect association within the inverse cross-sectional association linking physical exercise to depressive symptoms among rural older adults in China.

Figure 1 depicts the hypothesized pathways.

**Figure 1:**
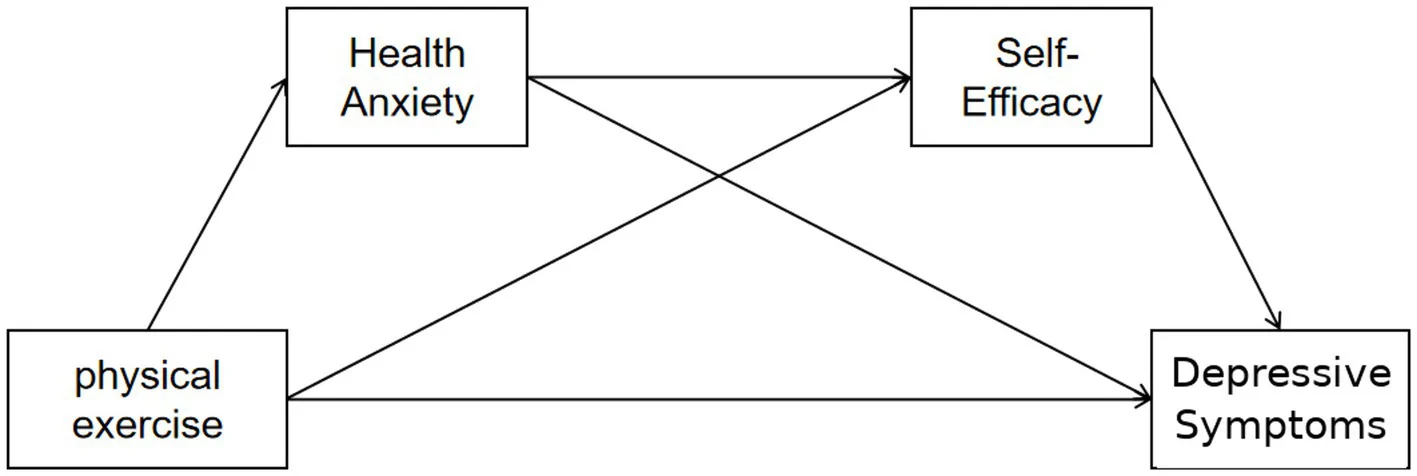
Theoretical model.

## 3 Methods

### 3.1 Study design

This study used a cross-sectional observational design to examine the associations among physical exercise, health anxiety, self-efficacy, and depressive symptoms in rural older adults in China. The protocol and reporting were conducted in accordance with applicable local regulations and the Declaration of Helsinki. Because the design was cross-sectional, all analyses were interpreted as associations and statistical indirect associations rather than causal effects or temporal mediation.

### 3.2 Study setting and data collection period

Data were collected from June to October 2025 in nine provincial-level administrative regions representing eastern, central, and western China, according to the three-region economic-geographic classification used by the National Bureau of Statistics of China. The eastern stratum included Shandong, Zhejiang, and Fujian; the central stratum included Henan, Hubei, and Anhui; and the western stratum included Sichuan, Shaanxi, and Gansu. Survey sites were administrative villages in predominantly agricultural counties; urban districts and highly urbanized suburban villages were not included. The selected villages had permanent older adult residents and household-registration rosters.

### 3.3 Sampling strategy

A combined multistage stratified random-sampling and quota-sampling design was used. The target population comprised rural permanent residents aged 60–69 years in the selected study areas. Sampling proceeded through the following stages:

1. Sampling-frame construction. The sampling frame was based on the 7th National Population Census microdata for rural permanent residents aged 60–69 years in mainland China. The frame was stratified into eastern, central, and western regions according to the national classification criteria.
2. Provincial sampling. Three provinces were selected by simple random sampling within each regional stratum, producing nine provinces in total.
3. Township and village sampling. Within each selected province, agricultural counties were grouped into high-, medium-, and low-per-capita-GDP tiers. One county was randomly selected from each tier; two townships were randomly selected within each sampled county; and two administrative villages with complete population registers were randomly selected within each township. The resulting field sample comprised 108 villages in 54 townships across 27 counties.
4. Individual sampling. For each selected village, a roster of residents aged 60–69 years was obtained from village committee household-registration records. Eligible residents were randomly selected from the roster as potential participants.
5. Quota implementation. Prespecified quotas for the 60–62, 63–65, and 66–69 age groups, cross-classified by sex, were used to approximate the age-sex composition of the rural older population reported in the 7th National Population Census. Recruitment within each village continued until the relevant quotas were reached.

Because participants were sampled within villages, observations could be correlated within the same village. All regression and indirect-association analyses therefore used village-level cluster-robust standard errors to account for potential within-village dependence.

### 3.4 Inclusion and exclusion criteria

Eligibility criteria were specified before fieldwork. Potential participants were screened at the household level against the inclusion criteria and then assessed against the exclusion criteria.

#### 3.4.1 Inclusion criteria

Participants were eligible for inclusion if they met all of the following conditions:

1. Participants were eligible if they were aged 60–69 years and had rural household registration or had lived permanently in the surveyed village for at least 12 consecutive months; provided written informed consent; and agreed to complete the four core scales and demographic questionnaire.
2. Had intact basic hearing, vision, and verbal communication abilities, and could independently understand questionnaire items with simple verbal prompts and complete self-reported scale assessments;
3. Provided written informed consent voluntarily after being clearly informed of the study purpose, procedures, anonymous data processing rules, and absence of adverse risks;
4. Agreed to complete all four core measurement scales and the demographic information sheet.

#### 3.4.2 Exclusion criteria

Participants who met all inclusion criteria were excluded from the final sample if any of the following conditions applied:

1. Participants were excluded if they had a documented severe cognitive disorder, Alzheimer’s disease, severe mental disorder, acute critical illness, advanced malignant tumor, or severe cardiovascular/cerebrovascular disease that prevented stable completion of the scales; had used anxiolytic or antidepressant medication regularly, received formal psychological counselling, antidepressant pharmacotherapy, or a standardized long-term exercise intervention during the preceding three months; showed poor response quality, including excessive identical responding, unresolved logical contradictions, more than 5% item-level missingness, a completely blank core scale, or completion in less than 10 min; withdrew before completing the core scales; or were judged by investigators to have deliberately concealed relevant health information or provided unreliable responses.
2. Had used anti-anxiety or anti-depressant psychotropic medications regularly within the 3 months prior to the survey, or had received formal psychological counseling, antidepressant pharmacotherapy, or standardized long-term exercise interventions organized by medical institutions in the same period;
3. Exhibited poor response quality: more than two-thirds of consecutive items in any single scale were filled with identical options (indicating perfunctory responding); obvious logical contradictions between reverse-scored and positively-worded items with no reasonable explanation after follow-up inquiry; missing items accounting for more than 5% of total questionnaire items, or any of the four core scales was completely blank; total survey completion time was shorter than the pre-specified minimum reasonable duration (10 min), judged as hasty and incomplete response by investigators;
4. Withdrew from the survey halfway without completing all core scales; or investigators recorded obvious emotional resistance or deliberate concealment of true health status during the interview, resulting in untrustworthy self-reported data.

### 3.5 Data collection procedure

All investigators received standardized training in the protocol, questionnaire administration, and communication with older adults before fieldwork. Face-to-face questionnaires were administered during home visits. Investigators provided standardized oral explanations and simple comprehension prompts without changing item meaning. Completed questionnaires were checked on site, and ambiguous or missing responses were confirmed with participants when possible.

### 3.6 Covariate selection and control strategy

Potential confounders were selected *a priori* using substantive relevance to both physical exercise and depressive symptoms, together with availability in the study dataset. The candidate set comprised age, sex, and region. Age was coded as a continuous covariate when used in models; sex and region were entered as categorical covariates using indicator coding. The same adjustment set—age, sex, and region—was included in the primary outcome regression and in each equation of the indirect-association models. No other demographic, socioeconomic, health-status, or village-level variables were available in the supplied analytic file for confounder adjustment.

### 3.7 Measures

The four core scales were administered to every participant in the same fixed order: the Physical Activity Rating Scale (PARS-3), the 10-Item Center for Epidemiologic Studies Depression Scale (CES-D 10), the Short Health Anxiety Inventory (SHAI), and the General Self-Efficacy Scale (GSE). Questionnaire order was not counterbalanced across participants.

#### 3.7.1 Physical Activity Rating Scale (PARS-3)

The validated Chinese version of the Physical Activity Rating Scale (PARS-3), revised for Chinese populations by Liang (1994), measured self-reported physical exercise. It assesses exercise intensity, frequency, and duration on 5-point response scales. The total score is calculated as exercise volume = frequency × (duration − 1) × intensity, ranging from 0 to 100. In the supplied data, the variable X represents the continuous total PARS-3 score.

For descriptive presentation only, the continuous PARS-3 score was categorized as low (≤19), moderate (20–42), or high (43–100) exercise volume. The categorical variable was not used in the primary regression or indirect-association models. Primary analyses retained the continuous X score to preserve information and estimate the association per one-point increase in exercise volume. Accordingly, “physical exercise” refers to the continuous PARS-3 score in inferential analyses, whereas “exercise-volume category” refers only to the descriptive classification.

In the present sample, the PARS-3 showed a KMO value of 0.705 and Bartlett’s test of sphericity was significant (681.895, *p* < 0.001). Cronbach’s α was 0.790.

#### 3.7.2 10-item Center for Epidemiologic Studies Depression Scale (CES-D 10)

The Chinese 10-item short form of the Center for Epidemiologic Studies Depression Scale (CES-D 10) assessed depressive symptom severity (Sheung-Tak, 2005). The scale contains two positively worded reverse-scored items and eight negatively worded items, rated from 0 to 3. Total scores range from 0 to 30, with higher scores indicating more severe depressive symptoms. The outcome was interpreted as a symptom score and not as a clinical diagnosis of depression.

In the present sample, the CES-D 10 showed a KMO value of 0.910 and a significant Bartlett’s test of sphericity (1591.159, *p* < 0.001). Cronbach’s α was 0.816.

#### 3.7.3 Short Health Anxiety Inventory (SHAI)

The Short Health Anxiety Inventory (SHAI) developed by Salkovskis et al. (2002), using the Chinese version evaluated by Zhang et al. (2015), assessed health anxiety. The 18-item scale covers worry about the likelihood of illness and fear of illness consequences. Total scores range from 18 to 90, with higher scores indicating greater health anxiety.

In the present sample, the SHAI showed a KMO value of 0.942 and a significant Bartlett’s test of sphericity (2354.200, *p* < 0.001). Cronbach’s α was 0.851.

#### 3.7.4 General Self-Efficacy Scale (GSE)

The General Self-Efficacy Scale (GSE) assessed general self-efficacy (Luo and Xie, 2023). It contains 10 items rated from 1 to 4, with total scores ranging from 10 to 40; higher scores indicate stronger perceived self-efficacy.

In the present sample, the GSE showed a KMO value of 0.904 and a significant Bartlett’s test of sphericity (1439.719, *p* < 0.001). Cronbach’s α was 0.803.

### 3.8 Data processing and analysis

Analyses were conducted in SPSS 27.0. The PROCESS macro version 4.0 was used for the indirect-association models. All tests were two-sided with α = 0.05. The analytic sample consisted of complete cases (*n* = 772).

Data preparation. Item responses were scored according to the instruments’ scoring rules, and total scores were calculated for PARS-3, CES-D 10, SHAI, and GSE. Descriptive statistics were calculated for the continuous variables and frequency distributions for the categorical variables.

Measurement and data-quality checks. Internal consistency was evaluated using Cronbach’s α. The manuscript also reports Harman’s single-factor test as a *post hoc* check of potential common-method variance. This test was treated as a diagnostic indicator rather than proof that common-method bias was absent.

Descriptive and regression analyses. Pearson correlations were used to summarize pairwise associations. The primary multivariable linear regression modeled CES-D 10 score as the outcome and included continuous PARS-3 score, SHAI score, GSE score, age, sex, and region as predictors. Thus, the six predictors used in the *a priori* power analysis correspond to the three focal variables plus the three prespecified covariates. This resolves the distinction between the power-analysis specification and the indirect-association equations, which were estimated separately.

Assumption assessment. Linearity, multicollinearity, residual behavior, homoscedasticity, and influential observations were assessed before interpreting the linear regression estimates. Tolerance and VIF were used to assess multicollinearity. Because the data were sampled within villages, standard errors were intended to be adjusted for village-level clustering; the reproducibility limitation concerning the unavailable cluster identifier is noted above.

Indirect-association analysis. A serial indirect-association model specified physical exercise (continuous PARS-3 score) as X, health anxiety as M1, self-efficacy as M2, and depressive symptom score as Y, with the hypothesized order X → M1 → M2 → Y. The model estimated the total association, the direct association, the specific indirect association through M1, the specific indirect association through M2, and the serial indirect association through M1 and M2. Age, sex, and region were included as covariates in each relevant regression equation. Indirect effects were evaluated with 5,000 nonparametric bootstrap resamples and bias-corrected and accelerated 95% confidence intervals. Coefficients were reported as unstandardized unless otherwise noted; standardized coefficients were reported only for the multiple linear regression. Village-level cluster-robust standard errors were used in the regression and indirect-association analyses, with the village as the clustering unit.

## 4 Results

### 4.1 Characteristics of the study population

The *a priori* power analysis for the multivariable linear regression model was conducted using G*Power 3.1. The analysis used a two-tailed α of 0.05, 80% power, an f^2^ of 0.02, and six predictors (physical exercise, health anxiety, self-efficacy, age, sex, and region). The minimum required sample size was 395, which was exceeded by the final analytical sample.

Most invited individuals completed the questionnaire, and the majority remained eligible for analysis after screening. Recruitment counts and response rates are presented in Table 1.

**Table 1:** Participant recruitment flow and response rates across stages.

Recruitment phase	Number	Percentage (%)
Individuals invited to participate	900	100.00
Did not participate / refused	28	3.11
Completed the questionnaire	872	96.89
Excluded after eligibility screening	100	11.11
Final eligible sample included in analysis	772	85.78

Percentages for non-participation, completion, and exclusion are based on 900 invited individuals; the final-sample percentage is 772/900.

The sample was broadly balanced by sex and region, with slightly more women than men. The youngest age group was less represented than the two older groups (Table 2).

**Table 2:** Demographic characteristics of the participants.

Variable	Category	Frequency	Percentage (%)
Age	60 ~ 62	221	28.6
	63 ~ 65	273	35.4
	66 ~ 69	278	36.0
Gender	Male	367	47.5
	Female	405	52.5
Region	Eastern	261	33.8
	Central	244	31.6
	Western	267	34.6

### 4.2 Data quality and statistical analysis

#### 4.2.1 Missing data handling

Questionnaires with more than 5% of total items missing or with any core scale (PARS-3, CES-D 10, SHAI, or GSE) entirely uncompleted were excluded. No missing values were present for the study variables or covariates among the 772 participants retained for analysis.

All analyses used complete cases. The number of observations was 772 for the descriptive, correlation, regression, and serial indirect-association analyses; no imputation was performed.

#### 4.2.2 Common method bias assessment

In Harman’s single-factor test, the first unrotated principal component accounted for 20.26% of the total variance.

### 4.3 Correlation analysis of research variables

Physical exercise was significantly associated with fewer depressive symptoms, lower health anxiety, and higher self-efficacy. Health anxiety was positively correlated with depressive symptoms and negatively correlated with self-efficacy, while self-efficacy was inversely correlated with depressive symptoms. Descriptive statistics and correlation coefficients are reported in Table 3.

**Table 3:** Means, standard deviations and correlation coefficients of all research variables.

Variable	Mean	SD	Physical exercise	Depressive symptoms	Health anxiety	Self-efficacy
Physical exercise	26.22	21.99	1			
Depressive symptoms	19.89	6.41	−0.272^***^	1		
Health anxiety	44.70	9.78	−0.394^***^	0.345^***^	1	
Self-efficacy	28.43	6.25	0.322^***^	−0.305^***^	−0.356^***^	1

*p < 0.05; **p < 0.01; ***p < 0.001.

The adjusted regression model was statistically significant (adjusted R^2^ = 0.166). Physical exercise and self-efficacy were each inversely associated with depressive symptom scores, whereas health anxiety was positively associated with these scores. All focal associations remained statistically significant after adjustment for age, sex, and region (Table 4).

**Table 4:** Multiple linear regression estimates for depressive symptom scores among rural older adults in China.

Variable	B	SE	95% CI	β	t	*P*	Tol.	VIF
Constant	19.399	1.754	[15.961, 22.837]	–	11.061	<0.001	–	–
Physical exercise	−0.036	0.011	[−0.058, −0.014]	−0.122	−3.336	<0.01	0.807	1.239
Health anxiety	0.151	0.024	[0.104, 0.198]	0.231	6.231	<0.001	0.786	1.272
Self-efficacy	−0.188	0.037	[−0.261, −0.115]	−0.183	−5.089	<0.001	0.835	1.198
Adjusted R^2^	–	–	–	–	–	<0.001	–	–
F	51.984	–	–	–	–	<0.001	–	–

B, unstandardized coefficient; SE, standard error; CI, confidence interval; β, standardized coefficient; Tol., tolerance; VIF, variance inflation factor. Estimates are adjusted for age, sex, and region and use village-level cluster-robust standard errors. *p* values are two-sided.

Regression diagnostics are summarized in Table 5. The reported tolerance, VIF, residual, homoscedasticity, and influence statistics did not indicate material violations of the assessed model assumptions. Regression estimates used village-level cluster-robust standard errors (Figure 2).

**Table 5:** Diagnostic statistics for multiple linear regression assumptions.

Assumption	Statistic
Normality of residuals	W = 0.990; JB = 20.095 (df = 2); K–S Z = 0.032; skew = −0.382, kurt = 0.206
Homoscedasticity	BP χ^2^ = 4.661 (df = 6); White χ^2^ = 34.929 (df = 27)
Absence of influential cases	Cook’s Dmax = 0.023; leverage max = 0.035; |DFFITS|max = 0.407
Independence of observations	DW = 1.327 (raw order); DW ≈ 2.00 after permutation (95% interval 1.86–2.14)

DW, Durbin–Watson; BP, Breusch–Pagan; JB, Jarque–Bera; K–S, Kolmogorov–Smirnov; DFFITS, difference in fits statistic.

**Figure 2:**
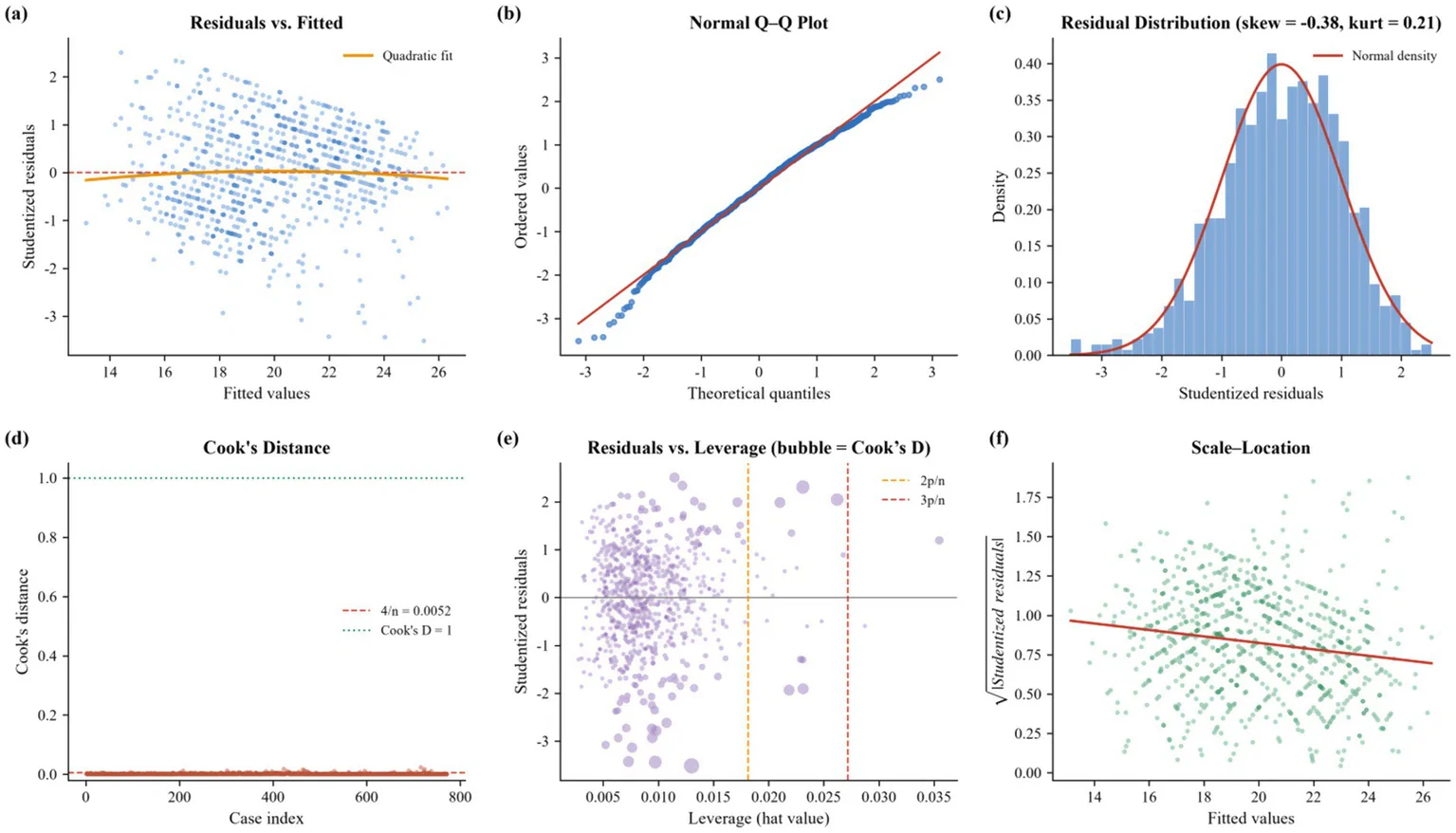
Regression diagnostic plots. **(a)** studentized residuals versus fitted values; **(b)** normal Q–Q plot; **(c)** distribution of studentized residuals; **(d)** Cook’s distance by case index; **(e)** residuals versus leverage (bubble size represents Cook’s distance); **(f)** scale–location plot.

### 4.4 Indirect-association analysis

The total association between physical exercise and depressive symptoms was negative, and the direct association remained significant after accounting for health anxiety and self-efficacy. The total indirect association was also negative and statistically significant (Table 6).

**Table 6:** Total, direct, and indirect associations in the serial indirect-association analysis.

Association type	Path	Estimate	SE	95% CI	Percentage (%)
Total association	Physical exercise → depressive symptoms	−0.0793	0.0101	[−0.0992, −0.0595]	100.00
Direct association	Physical exercise → depressive symptoms	−0.0356	0.0107	[−0.0566, −0.0147]	44.89
Total indirect association	Via health anxiety and self-efficacy	−0.0437	0.0056	[−0.0555, −0.0331]	55.11
Specific indirect association	Physical exercise → health anxiety → depressive symptoms	−0.0266	0.0046	[−0.0362, −0.0180]	33.54
Specific indirect association	Physical exercise → self-efficacy → depressive symptoms	−0.0115	0.0032	[−0.0183, −0.0060]	14.50
Serial indirect association	Physical exercise → health anxiety → self-efficacy → depressive symptoms	−0.0057	0.0014	[−0.0087, −0.0032]	7.07

CI, confidence interval; SE, standard error. Estimates are unstandardized and adjusted for age, sex, and region; village-level clustering was accounted for using cluster-robust standard errors. Percentages are calculated relative to the total association and may not sum exactly because of rounding.

The specific indirect associations through health anxiety and self-efficacy, as well as the serial indirect association through both constructs, were negative, with bootstrap confidence intervals excluding zero. The health-anxiety pathway had the largest estimated magnitude, whereas the serial pathway had the smallest. These findings were consistent with the hypothesized pattern of associations but do not establish temporal ordering or causal mediation (Figure 3).

**Figure 3:**
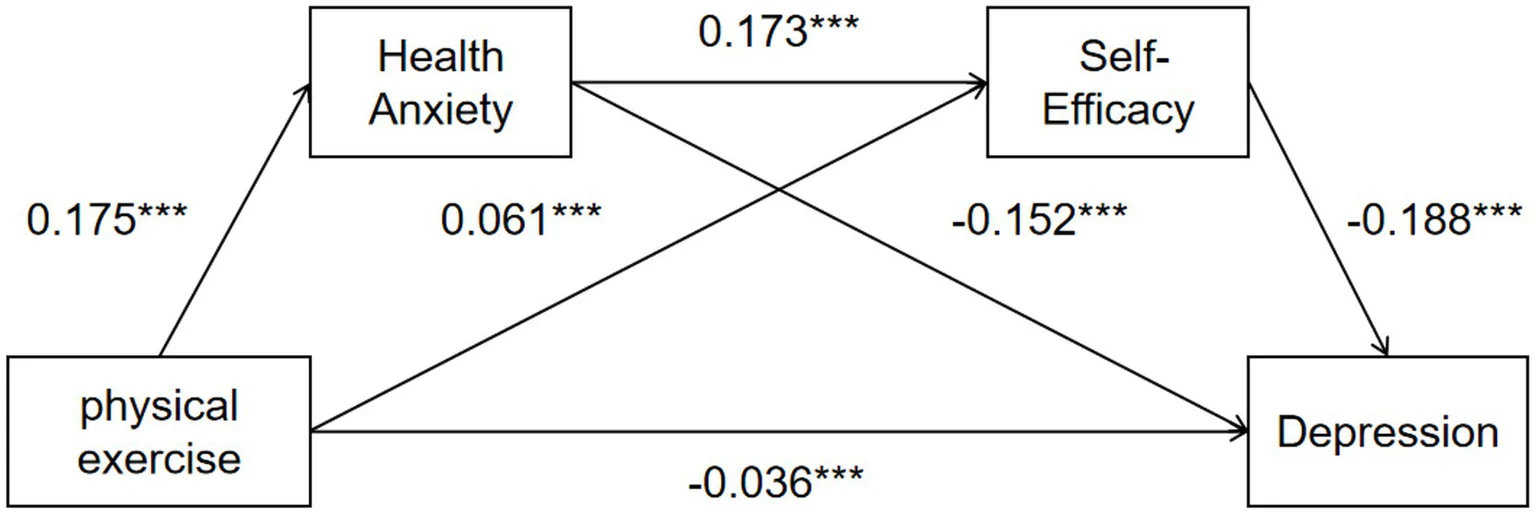
Serial indirect-association model of health anxiety and self-efficacy.

## 5 Discussion

In this sample of rural Chinese adults aged 60–69 years, higher physical exercise scores were associated with fewer depressive symptoms. The fitted model also showed specific statistical indirect associations through health anxiety and self-efficacy and a smaller serial indirect association through both constructs. These findings are consistent with the hypothesized pattern, but they do not establish that physical exercise changes these psychological characteristics or that changes in them precede changes in depressive symptoms.

The inverse association is broadly consistent with research on exercise and depressive symptoms in older adults (Lili et al., 2024). Research in rural China has also examined depressive symptoms in relation to the distribution of sleep, sedentary behavior, and physical activity across the day (Zhao et al., 2024). Those measures describe a broader behavioral pattern than the PARS-3 exercise score used here. The present analyses do not establish whether the observed association is independent of agricultural work or other daily physical activity, nor whether purposeful exercise is superior to productive labor.

The specific indirect association through health anxiety was larger in the fitted model than the other specific indirect associations. This pattern warrants attention to health-related worry as a correlate of depressive symptoms. The interpretation of bodily sensations as health threats provides a conceptual basis for considering this construct (Asmundson et al., 2010). However, the observed association does not demonstrate that exercise reduces health anxiety or that health anxiety subsequently changes depressive symptoms. Its relative magnitude does not establish causal importance or intervention priority.

The specific indirect association through self-efficacy similarly suggests that perceived coping capacity is relevant to the observed exercise–symptom association. Exercise participation and confidence in coping may be related in either direction, while depressive symptoms may also be associated with both. The current data cannot distinguish these possibilities or establish self-efficacy as a mechanism of exercise-related change.

The serial indirect association was the smallest of the three specific indirect associations. Its statistical significance should not be equated with clinical importance, and no clinical threshold was evaluated. Because all constructs were measured at the same time, the ordering of health anxiety before self-efficacy is assumed rather than demonstrated. The finding therefore offers a hypothesis for prospective testing, without supporting a sequential psychological mechanism or a staged intervention strategy.

### 5.1 Limitations

The cross-sectional design cannot establish temporal ordering or causal mediation. Reverse causation is plausible: depressive symptoms may be associated with less exercise, greater health anxiety, and lower self-efficacy. Residual confounding by factors such as socioeconomic circumstances, physical health, and social support may also influence the associations; the reported adjustment does not eliminate these concerns; Since the questionnaires were distributed to all participants in the same sequence, the effects of practice and fatigue were not controlled, which may influence the research results.

All core variables were self-reported during the same interview, creating potential recall, social-desirability, and common-method bias. CES-D 10 scores assess depressive symptoms rather than clinical depression. Participants were nested within villages, and the extent of within-village dependence was not quantified. Incomplete reporting of the clustering procedure, particularly for indirect-association confidence intervals, limits assessment of statistical precision; the robustness of the reported significance to clustering therefore remains uncertain.

Generalizability is limited to the sampled rural adults aged 60–69 years and may be affected by participation and exclusion criteria. The analysis of an overall exercise score does not resolve differences by exercise type or setting. Other relevant correlates and potential pathways, including sleep, social interaction, and biological factors, were not comprehensively assessed.

### 5.2 Future directions

Future research should prioritize multi-wave longitudinal studies that measure all four constructs repeatedly to evaluate temporal ordering and bidirectional associations. Randomized exercise trials with repeated assessments of health anxiety, self-efficacy, and depressive symptoms could test whether exercise changes these outcomes and whether psychological changes precede symptom changes. Such studies should prespecify confounder control and account for village-level clustering. Objective movement measures and broader sampling would help address measurement limitations and assess generalizability.

## 6 Conclusion

Among rural Chinese adults aged 60–69 years, higher physical exercise scores were associated with fewer depressive symptoms. Health anxiety and self-efficacy were involved in specific statistical indirect associations, including a smaller serial indirect association. These findings describe cross-sectional associations and statistical indirect effects; they do not establish causality or temporal mediation.

## Data Availability

The original contributions presented in the study are included in the article/supplementary material, further inquiries can be directed to the corresponding author.
